# MONOPTEROS directly activates the auxin-inducible promoter of the Dof5.8 transcription factor gene in *Arabidopsis thaliana* leaf provascular cells

**DOI:** 10.1093/jxb/eru418

**Published:** 2014-10-21

**Authors:** Mineko Konishi, Tyler J. Donner, Enrico Scarpella, Shuichi Yanagisawa

**Affiliations:** ^1^Biotechnology Research Center, The University of Tokyo, Yayoi 1-1-1, Bunkyo-ku, Tokyo 113-8657, Japan; ^2^Department of Biological Sciences, University of Alberta, CW-405 Biological Sciences Building, Edmonton, Alberta, Canada T6G 2E9

**Keywords:** *Arabidopsis thaliana*, auxin response, Dof transcription factor, embryo development, MONOPTEROS, vascular development.

## Abstract

MONOPTEROS (MP) is an auxin-responsive transcription factor that is required for primary root formation and vascular development, whereas Dof5.8 is a Dof-class transcription factor whose gene is expressed in embryos as well as the pre- and procambial cells in the leaf primordium in *Arabidopsis thaliana*. In this study, it is shown that MP directly activates the *Dof5.8* promoter. Although no apparent phenotype of the single *dof5.8* mutants was found, phenotypic analysis with the *mp dof5.8* double mutants revealed that mutations within *Dof5.8* enhanced the phenotype of a weak allele of *mp*, with an increase in the penetrance of the ‘rootless’ phenotype and a reduction in the number of cotyledons. Furthermore, interestingly, although *mp* mutants showed reduced vascular pattern complexity in cotyledons, the *mp dof5.8* double mutants displayed both more simplex and more complex vascular patterns in individual cotyledons. These results imply that the product of *Dof5.8* whose expression is regulated by MP at least in part might be involved in multiple processes controlled by MP.

## Highlight

An *Arabidopsis* gene, *Dof5.8*, is a target of the auxin-responsive MONOPTEROS transcription factor. Mutations in *Dof5.8* interfere with embryo development and vein patterning when combined with a *monopteros* allele.

## Introduction

MONOPTEROS (MP), which is also known as auxin response factor 5 (ARF5) of the ARF family, is a key regulator that functions in the establishment of vasculature and body patterns in embryonic and post-embryonic development in *Arabidopsis thaliana* (Hardtke and Berleth, 1998; Ulmasov *et al*., 1999*a*, *b*). Mutations within the *MP* gene interfere with the body axis patterning in the early embryo and the formation of vascular strands. Thus, the *mp* mutants show reduced complexity in vascular patterns in both cotyledons and true leaves, and the *mp* seedlings are often rootless and have only one cotyledon (Berleth and Jürgens, 1993; Przemeck *et al.*, 1996). Because MP is a transcription factor, direct target genes of MP have been searched for in order to clarify MP-mediated regulations. Consistent with the diverse functions of MP in embryonic root initiation, lateral organ initiation, shoot meristem cell regulation, and vascular patterning in leaves (Berleth and Jürgens, 1993; Przemeck *et al.*, 1996; Hardtke *et al.*, 2004; Schuetz *et al.*, 2008), recent intensive studies revealed that MP activates the expression of *DRN* involved in cell patterning in embryos (Cole *et al.*, 2009), *TMO* genes crucial for embryonic root initiation (Schlereth *et al.*, 2010), and *LFY* for flower initiation (Yamaguchi *et al.*, 2013), and represses the expression of *ARR7* and *ARR15*, negative regulators of cytokinin signalling, in the shoot apical meristem (Zhao *et al.*, 2010). Furthermore, it has been reported that MP regulates the expression of *BRX* involved in cross-talk between the auxin and brassinosteroid pathways (Bauby *et al.*, 2007; Beuchat *et al.*, 2010; Scacchi *et al.*, 2010), *Athb8* associated with the formation of vascular strands in leaves (Donner *et al.*, 2009), and *MP* itself (Lau *et al.*, 2011). Target genes of MP probably vary depending on organ, tissue type, or developmental stage, and thus MP functions and MP-induced regulation still remain largely unknown.

Dof transcription factors are a family of transcription factors that harbour a plant-specific Dof DNA-binding domain that recognizes 5ʹ-AAAG-3ʹ or 5ʹ-CTTT-3ʹ motifs (Yanagisawa, 2002, 2004). Although the physiological functions of Dof transcription factors are highly diverse, many *Dof* genes are expressed in the vasculature or during vascular development (Gualberti *et al.*, 2002; Imaizumi *et al.*, 2005; Skirycz *et al.*, 2006; Konishi and Yanagisawa, 2007; Guo *et al.*, 2009; Gardiner *et al.*, 2010; Schlereth *et al.*, 2010; Le Hir and Bellini, 2013). It was shown previously that the promoter of *A. thaliana Dof5.8* is specifically active in embryos during the transition and heart stages and the future vasculature of cotyledons at the walking-stick stage, as well as procambial cells (vascular precursors) and pre-procambial cells (cells in the middle of the first stage of vascular development from the ground meristem cells to the procambial cells) in the leaf primordium (Konishi and Yanagisawa, 2007).

As the initial steps of vascular development in leaves in dicots are triggered by auxin flow, and then auxin-induced MP activity modulates gene expression for formation of the vascular network (Donner *et al.*, 2009; Ckurshumova *et al.*, 2011), it is known that pre-procambial and procambial cells (hereafter collectively termed ‘provascular cells’) are characterized by expression of the auxin-responsive marker gene, *DR5:GUS* (Mattsson *et al.*, 2003), or the auxin efflux carrier protein, PIN1 (Scarpella *et al.*, 2006; Wenzel *et al.*, 2007). Based on the expression pattern of *Dof5.8* in embryos and provascular cells in the leaf primordium, we speculated that *Dof5.8* might be a target of MP and associated with MP-regulated processes. To examine this hypothesis, molecular genetic and biological analyses were performed in this study. The results indicate that MP directly activates the *Dof5.8* promoter whereas mutations within *Dof5.8* influence multiple phenotypes of the *mp* mutant, *arf5-2*.

## Materials and methods

### Plant materials

*Arabidopsis thaliana* ecotype Columbia (Col) was used as the wild-type strain in all experiments. Seeds of the *mp* mutants, *arf5-1*, SALK_001058 and *arf5-2* (also called *mp-S319* or SALK_021319), and SALK T-DNA lines of *Dof5.8* were obtained from the Arabidopsis Resource Center (Alonso *et al.*, 2003; Okushima *et al.*, 2005; Donner *et al.*, 2009). DR5-GUS (β-glucuronidase) seeds (Ulmasov *et al.*, 1997*b*) were a gift from Dr Tom J. Guilfoyle. For the analysis of transcript levels of *Dof5.8* in *mp* alleles, selfed seeds from heterozygous *mp* plants were sown. Seedlings exhibiting the rootless phenotype were collected for quantitative reverse transcription–polymerase chain reaction (qRT–PCR) analysis. To generate the double mutants of *arf5-2* and *dof5.8-1* or *dof5.8-2*, the *dof5.8* plants that are homozygous for a T-DNA insertion were crossed to heterozygous *arf5-2* plants, and F_2_ plants homozygous for the *dof5.8* T-DNA allele and heterozygous for *arf5-2* allele were selected by PCR-based genotyping. For phenotypic analysis, rootless F_3_ seedlings, which are homozygous for the *arf5-2* allele (Table 1), were picked for analysis of cotyledon numbers and vascular patterns. For the analysis of the *Dof5.8* promoter activity in the *arf5-2* background, the Dof5.8pro-GUS line harbouring the GUS reporter gene under the control of the *Dof5.8* promoter (Konishi and Yanagisawa, 2007) was crossed to the *arf5-2* heterozygous plant. The F_3_ population that was homozygous for the Dof5.8pro-GUS transgene linked to the glufosinate ammonium resistance gene and heterozygous for the *arf5-2* allele was selected by phenotypic analysis of the glufosinate ammonium resistance and rootless phenotype or genotyping using a cotyledon of F_3_ seedlings.

**Table 1.** Segregation of the *arf5-2* allele among populations derived from plants heterozygous for the *arf5-2* allele in the wild-type, *dof5.8-1* or *dof5.8-2* background

Genotype of parental plant	No. of seedlings with the indicated genotype at the *MP* locus (% of total)				No. of rootless seedlings^*a*^
	*MP/MP*	*MP/arf5-2*	*arf5-2/arf5-2*	Total	
*MP/arf5-2*	31 (35.2%)	38 (43.2%)	19 (21.6%)	88	2
*dof5.8-1/dof5.8-1; MP/arf5-2*	26 (26.8%)	51 (52.6%)	20 (20.6%)	97	14
*dof5.8-2/dof5.8-2; MP/arf5-2*	23 (19.3%)	69 (58.0%)	27 (22.7%)	119	15

^*a*^ All rootless seedlings were homozygous for the *arf5-2* allele.

### Plant growth conditions

Seeds were sterilized and sown on half-strength Murashige and Skoog (1/2MS) agar plates containing 1% sucrose, as described previously (Konishi and Yanagisawa, 2008). After 3–4 d of stratification, plates were transferred to a chamber set at 23 °C with continuous illumination (60 μE m^–2^ s^–1^). For 2,4-dichlorophenoxyacetic acid (2,4-D) treatment, seedlings were grown in liquid 1/2MS medium for 3 d and treated or not with 10 μM 2,4-D for 16h. For the analysis of the vascular pattern, seeds were plated on 1/2MS agar medium containing 1% sucrose, solidified with 0.3% agar. For protoplast transient assays, ecotype Col plants were grown on peat containing nutrients (Sakatanotane Co., Yokohama, Kanagawa, Japan) at 23 °C for 3 weeks under continuous light.

### Genotyping

DNA extraction was performed according to Konishi and Sugiyama (2003). Primers used in PCR are listed in Supplementary Table S1 available at *JXB* online.

### Protoplast transient assays

The DNA fragment from the *Dof5.8* promoter was amplified by PCR (Konishi and Yanagisawa, 2007), and used to replace the *Cauliflower mosaic virus* 35S RNA promoter in pJD301 (Luehrsen *et al.*, 1992) to produce reporter plasmids containing the luciferase (LUC) gene. The deleted versions of the *Dof5.8* promoter were generated by digestion of the full-length promoter fragment with *Sph*I for truncation at position –1301 (relative to the translation start site) and *Eco*RI for truncation at position –1077, whereas mutated *Dof5.8* promoters were generated by PCR-based mutagenesis, as described previously (Konishi and Yanagisawa, 2010). Primers used for the mutagenesis are listed in Supplementary Table S1 at *JXB* online. For the construction of effector plasmids, *MP* or the *BODENLOS* (*BDL*) cDNA insert was amplified by RT–PCR and inserted in place of *EIN3* cDNA in the 35SC4PPDK-EIN3-MYC plasmid (Yanagisawa *et al.*, 2003). All constructs were verified by DNA sequencing. Co-transfection of reporter and effector plasmids and an internal control plasmid (UBQ10-GUS) into *A. thaliana* mesophyll protoplasts was carried out according to the method of Yoo *et al.* (2007). Measurement of LUC and GUS activities and calculations of relative LUC activity levels were performed as described previously (Yanagisawa *et al.*, 2003). For auxin treatment, protoplasts were incubated in the absence or presence of 1 μM indole-3-acetic acid (IAA) after co-transfection.

### qRT–PCR analysis

RNA preparation and qRT–PCR were performed as described previously (Konishi and Yanagisawa, 2010). The primers used are listed in Supplementary Table S1 at *JXB* online.

### Construction of binary plasmids and generation of transgenic *A. thaliana* plants

To construct binary vectors for GUS staining, the DNA fragments for the truncated or mutated *Dof5.8* promoters were excised from the respective reporter plasmids used in protoplast transient assays and then inserted in place of the *Dof5.8* promoter in the pCB-Dof5.8pro-GUS construct. The transformations of *A. thaliana* were carried out using these binary vectors, as described previously (Konishi and Yanagisawa, 2007).

### GUS staining and histological analysis

Histochemical GUS staining was essentially performed as described previously (Konishi and Yanagisawa, 2007). Samples were fixed in 90% acetone at –20 °C, rinsed four times with 0.1M sodium phosphate buffer (pH 7.4), and then incubated in X-Gluc solution [0.1M sodium phosphate (pH 7.4), 3mM potassium ferricyanide, 0.5mM potassium ferrocyanide, 0.5g l^–1^ 5-bromo-4-chloro-3-indolyl-β-d-glucuronide cyclohexilammonium salt] at 37 °C. When GUS activity was weak, the concentration of potassium ferricyanide was reduced according to Donnelly *et al.* (1999). Potassium ferricyanide at 1.75mM was thus used for the *Dof5.8* promoter truncated at –1301 and the promoter containing a 486bp fragment (from –1558 to –1073) of the *Dof5.8* promoter upstream of the 35S minimal promoter, and 0.5mM potassium ferricyanide was used for the *Dof5.8* promoter truncated at –1077. After staining, samples were incubated in methanol to remove chlorophyll and then mounted in the clearing solution (a mixture of chloral hydrate, water, and glycerol in a ratio of 8:2:1). Observation was performed using a stereomicroscope (MZ16F, Leica Microsystems, Germany) or a microscope equipped with Nomarski optics (BX51, Olympus Co., Tokyo, Japan). For the observation of vascular patterns, cotyledons were fixed in a mixture of ethanol and acetic acid in a ratio of 9:1, hydrated through a graded series of ethanol, and then mounted with the clearing solution (Konishi and Sugiyama, 2003).

### Chromatin immunoprecipitation analysis

Chromatin immunoprecipitation (ChIP) analysis was carried out to examine the binding of MP to the *Dof5.8* promoter, as described previously (Donner *et al.*, 2009).

## Results

### Regulation of the provascular expression of *Dof5.8* by MP

**Fig. 1.**
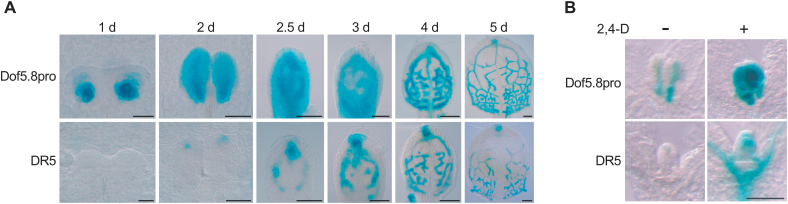
Provascular activity of the *Dof5.8* promoter and its enhancement by auxin. (A) Time course analysis of GUS expression under the control of the *Dof5.8* promoter (Dof5.8pro) or the DR5 element (DR5) during development of the primordia of the first true leaves. Two primordia flanking the shoot apical meristem (1 d and 2 d) and individual leaf primordia (2.5–5 d) are shown. Scale bars=20 μm in 1 d and 2 d, 50 μm in 2.5 d and 3 d, 100 μm in 4 d and 5 d. (B) Effect of auxin treatment on the activity of the *Dof5.8* promoter. Seedlings were treated or not with 10 μM 2,4-D for 16h. Scale bar=100 μm.

It was shown previously that the *Dof5.8* promoter is active in provascular cells in leaf primordia (Konishi and Yanagisawa, 2007). Because the synthetic auxin response element, DR5, induces a similar expression pattern in leaves (Ulmasov *et al.*, 1997*b*; Mattsson *et al.*, 2003; Scarpella *et al.*, 2006), the expression patterns produced by a GUS reporter gene under the control of either the *Dof5.8* promoter or DR5 during the development of the first leaf primordia were compared (Fig. 1A). The *Dof5.8* promoter initially directed strong GUS expression deep inside the bulges of the leaf primordia (Fig. 1A, ‘1d’). This expression then expanded vertically with the upward extension of the primordia. From day 2.5 onwards, reporter expression was localized to the provascular network in a pattern similar to that produced by DR5. This suggested that the activity of the *Dof5.8* promoter is regulated at least in part by auxin that accumulated during pre-procambium formation. Consistently, a treatment with the synthetic auxin 2,4-D significantly strengthened expression of the GUS reporter gene under the control of the *Dof5.8* promoter (Fig. 1B).

**Fig. 2.**
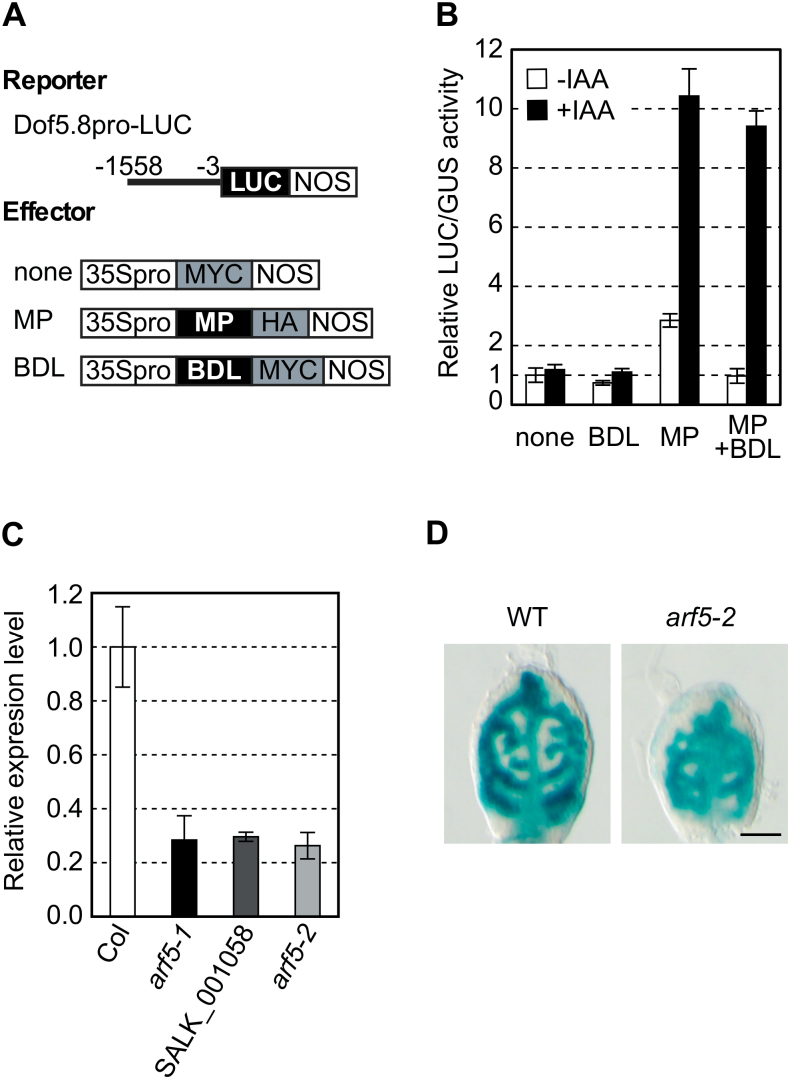
Activation of the *Dof5.8* promoter by MP. (A) Schematic diagrams of the reporter and effector constructs used in the protoplast transactivation assay described in (B). Numbers indicate nucleotide positions relative to the translation start codon. ‘MYC’ and ‘HA’ are MYC- and haemagglutinin-tag peptides. (B) Effects of MP and auxin on the activity of the *Dof5.8* promoter in protoplasts. The Dof5.8pro-LUC reporter construct was co-transfected with the expression plasmid of BDL, MP, or both, or an empty vector (none) and then protoplasts were incubated in the presence (+ IAA) or absence (– IAA) of 1 μM IAA. An internal control plasmid (UBQ10-GUS) was also co-transfected to normalize LUC reporter activity levels. Relative levels of LUC activity are shown as the means ±SD (*n*=3). (C) Relative *Dof5.8* transcript levels in three *mp* alleles. Total RNA was extracted from the shoots of 4-day-old seedlings and used for qRT–PCR analysis. The transcript levels in wild-type *A. thaliana* (Col) were set to 1, as all three *mp* alleles (*arf5-1*, SALK_001058, and *arf5-2*) were in the Colombia background. Data are shown as the means ±SD (*n*=3). (D) GUS staining of the first true leaves of 4-day-old wild-type and *arf5-2* seedlings that harbour the GUS gene under the control of the *Dof5.8* promoter. The *arf5-2* homozygous seedlings obtained from a segregating population of the plant homozygous for the Dof5.8pro-GUS transgene and heterozygous for the *arf5-2* allele were used. Scale bar=50 μm.

Among the ARFs, MP is known to be involved in vascular development. *mp* mutants show reduced vascular pattern complexity and sometimes form a disconnected vasculature (Berleth and Jürgens, 1993; Przemeck *et al.*, 1996; Hardtke and Berleth, 1998). Thus, it was hypothesized that the *Dof5.8* promoter is activated by MP. To examine this possibility, transactivation assays were performed in *A. thaliana* protoplasts using a reporter plasmid containing the LUC gene under the control of the *Dof5.8* promoter (Dof5.8pro-LUC) and an effector plasmid directing the constitutive high level expression of MP (Fig. 2A). The results indicated that MP increases *Dof5.8* promoter activity by ~3-fold and that the MP-induced activation was hampered by the co-expression of BDL/IAA12 (Fig. 2B), a cognate repressor protein of MP (Hamann *et al.*, 2002). Furthermore, the addition of a natural auxin, IAA, into the incubation buffer of the transfected protoplasts enhanced the effect of MP. This could be due to the activation of MP through the degradation of repressor proteins of MP, namely endogenous Aux/IAA proteins or co-expressed BDL, by auxin (Mockaitis and Estelle, 2008). These findings suggested that MP could transactivate the *Dof5.8* promoter in response to auxin.

To substantiate that the expression of *Dof5.8* is under the control of MP *in planta*, *Dof5.8* transcript levels were first analysed in three *mp* alleles by qRT–PCR. The expression levels of *Dof5.8* in all *mp* mutants, including *arf5-1* (Alonso *et al.*, 2003; Okushima *et al.*, 2005) and *arf5-2* (Alonso *et al.*, 2003; Donner *et al.*, 2009), were reduced to ~30% of those in the wild-type *A. thaliana* (Fig. 2C). Furthermore, when the GUS gene fused to the *Dof5.8* promoter was introduced into the *arf5-2* mutant by crossing, the GUS expression was found to be lower in leaf primordia of the *arf5-2* seedlings than in those of the wild-type seedlings (Fig. 2D). These results indicate that *Dof5.8* expression is regulated by MP activity, at least in part.

### Identification of the MP-binding sites required for provascular activation of the *Dof5.8* promoter

**Fig. 3.**
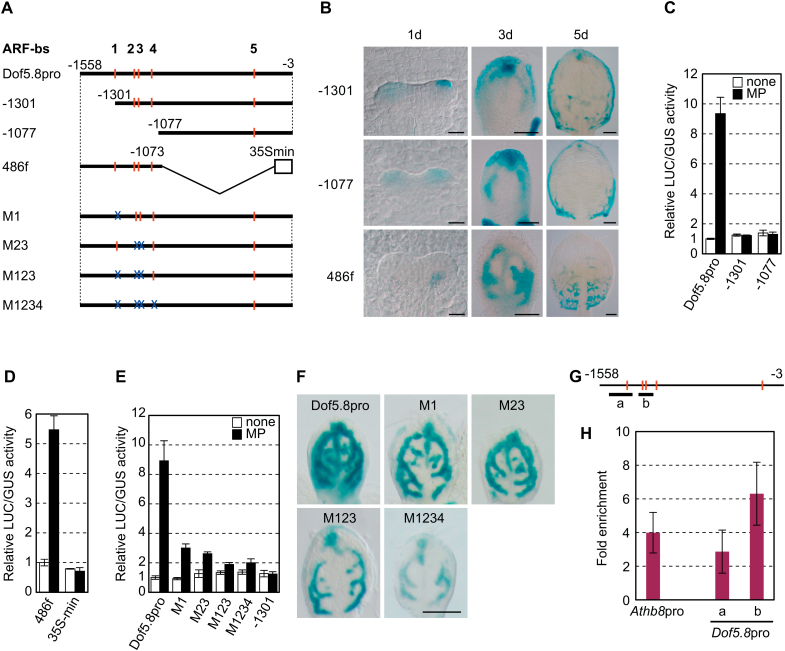
Identification of MP-binding sites and the promoter region required for *Dof5.8* expression in provascular cells. (A) Schematic representation of the promoter fragments used for deletion and mutational analyses of the *Dof5.8* promoter. The sequences matching the ARF-binding consensus sequence (ARF-bs), and disruptions in these sequences are indicated by red bars and blue ‘X’s, respectively. Numbers indicate nucleotide positions relative to the translation start codons. A 486bp fragment from –1558 to –1073 was fused to the 35S minimal promoter truncated at –72 (35S min) to generate a fusion promoter (the 486f promoter). The mutated sequences are 5’-ACAGAG-3’ in ARF-bs 1–3 and 5’-ACAGTG-3’ in ARF-bs 4. (B) The activity of the truncated *Dof5.8* promoters and the 486f promoter in the primordia of the first leaves. GUS staining of the first leaves of the transgenic seedlings carrying the GUS gene under the control of the deletion promoters described in (A). Scale bars=20 μm (1 d), 50 μm (3 d), and 100 μm (5 d). (C–E) MP-mediated transactivation of truncated *Dof5.8* promoters (C), the 486f synthetic promoter (D), and mutated *Dof5.8* promoters (E) in protoplasts. Promoters fused to LUC were co-transfected with the 35S-MP-HA plasmid (black bars) or an empty vector (white bars). Data are shown as the means ±SD (*n*=3). (F) GUS staining of the first leaves of the 4-day-old transgenic seedlings carrying the GUS gene under the control of the mutated promoters described in (A). Scale bar=100 μm. (G) Schematic representation of the *Dof5.8* promoter showing ARF-binding sites (red bars) and the positions of the amplified DNA fragments (bars a and b) used in the ChIP-qPCR analysis shown in (H). (H) ChIP analysis of the binding of MP to the *Dof5.8* promoter. Four-day-old transgenic seedlings expressing CFP-tagged MP were used. A DNA fragment from the *Athb8* promoter was amplified as a positive control (Donner *et al.*, 2009). The values were normalized using amplified DNA from a promoter unrelated to MP (the *UBQ10* promoter).

To determine the region within the *Dof5.8* promoter that is required for provascular expression, increasing segments of this promoter were deleted from its 5’ end (Fig. 3A). Deletion of the region from –1558 to –1301 in the *Dof5.8* promoter diminished GUS expression in provascular cells (Fig. 3B). In addition, a synthetic promoter in which the 486bp fragment from –1558 to –1073 was fused to the 35S minimal promoter (the 486f promoter) was able to direct provascular GUS reporter expression (Fig. 3A, B), indicating that this 486bp region is sufficient to confer *Dof5.8* expression in provascular cells.

The relationship between MP-mediated activation and expression of *Dof5.8* in provascular cells was examined using the series of truncated *Dof5.8* promoters. The results revealed that MP transactivated only the 486f synthetic promoter (Fig. 3C, D), indicating that the region from –1558 to –1301 is required, whilst the region from –1558 and –1073 is sufficient for both provascular cell expression and activation by MP (Fig. 3B–D). These data further indicate that an intimate relationship exists between MP-mediated activation and expression of *Dof5.8* in provascular cells.

ARFs are known to recognize and bind to 5ʹ-TGTCNC-3ʹ sequences (Ulmasov *et al.*,1997*a*, 1999b). Four putative ARF-binding sequences were identified in the region between positions –1558 and –1073 of the *Dof5.8* promoter (Fig. 3A). A mutation in site 1 (M1) or simultaneous mutations in sites 2 and 3 (M23) reduced the magnitude of activation by MP (Fig. 3A, E). Combination of these mutations (M123) led to an enhanced reduction in reporter enzyme activity, whereas disruption of the fourth site (M1234) had no apparent additional effect. The effects of these mutations on provascular expression in leaf primordia were also assessed (Fig. 3F). The mutated *Dof5.8* promoters still directed provascular expression in leaf primordia, but mutations significantly decreased the GUS expression levels. These results suggest that MP recognizes multiple sites in the *Dof5.8* promoter that are involved in provascular expression of *Dof5.8*, although other sites in addition to these putative ARF-binding sites analysed are probably involved in provascular expression of *Dof5.8*, as is argued in detail in the Discussion.

The binding of MP to the *Dof5.8* promoter *in vivo* was investigated using ChIP analysis of transgenic *A. thaliana* plants expressing a functional cyan fluorescent protein (CFP)-tagged MP under the control of its own promoter (Donner *et al.*, 2009). As shown in Fig. 3G and H, the results indicated that the binding of MP to the *Dof5.8* promoter is comparable with (amplicon ‘a’) and even stronger than (amplicon ‘b’) that to the *Athb8* promoter, a known target of MP during vascular development (Donner *et al.*, 2009). Collectively, these results suggest that the *Dof5.8* promoter is a direct target of MP in provascular cells and that the entire region from –1558 to –1077 contributes to provascular expression of *Dof5.8*.

### Loss of *Dof5.8* affects the root and cotyledon phenotypes of the *arf5-2* mutant

**Fig. 4.**
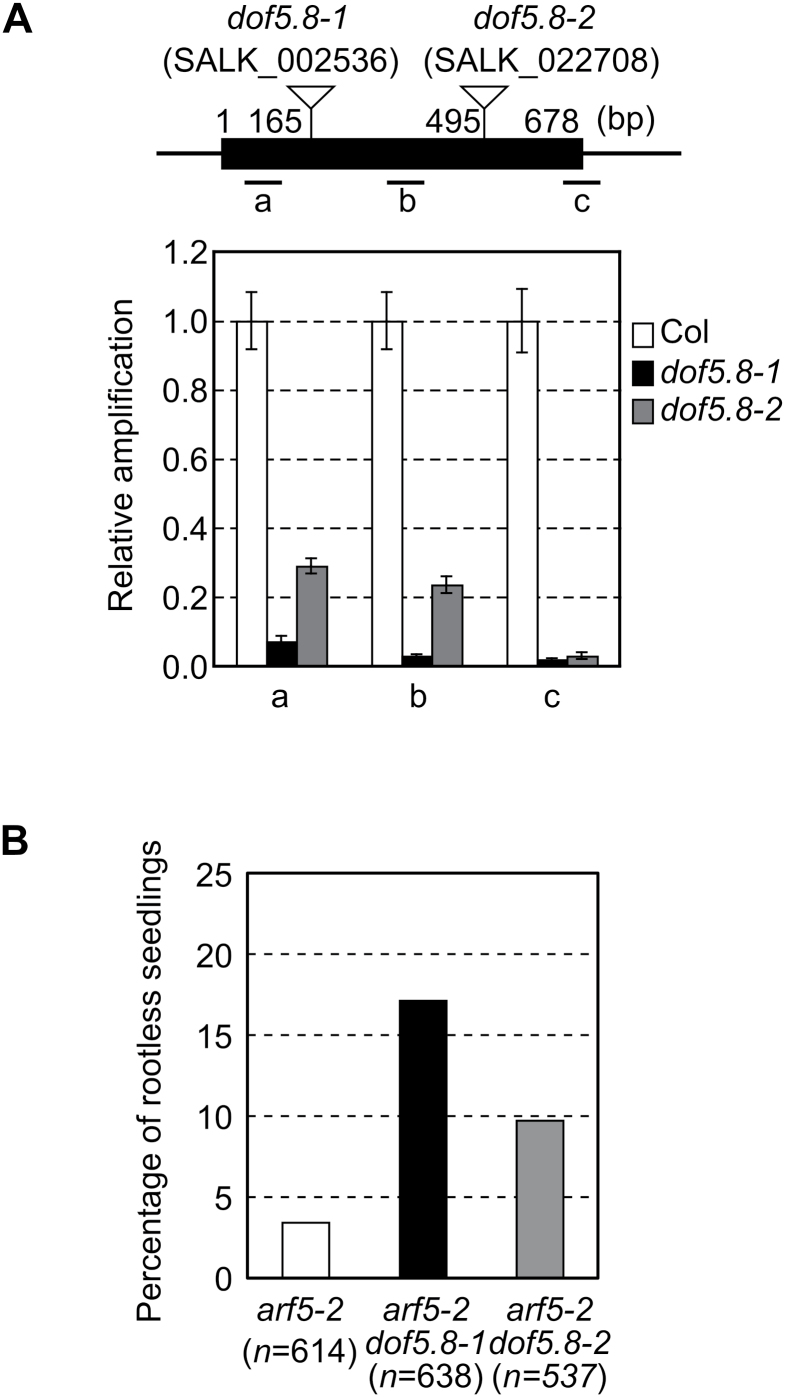
Enhancement of the rootless phenotype of the *arf5-2* mutant by *dof5.8* mutations. (A) The positions of T-DNA insertions and transcript levels of *Dof5.8* in the *dof5.8-1* and *dof5.8-2* mutant lines. The black box indicates the exon, and the positions of the three amplicons (a–c) are shown below. Nucleotide numbers are given relative to the translation start codon. The transcript levels in wild-type *A. thaliana* (Col) were set to 1. Data are shown as the means ±SD (*n*=3). (B) The percentages of the seedlings with the rootless phenotype from segregating populations of *arf5-2* single and *arf5-2 dof5.8* double mutants. Populations derived from parental plants heterozygous for the *arf5-2* allele in the wild-type, *dof5.8-1* homozygous or *dof5.8-2* homozygous background were analysed.

To explore the role of *Dof5.8*, two T-DNA insertion lines, *dof5.8-1* (SALK_002536) and *dof5.8-2* (SALK_022708), were analysed. Because T-DNA was inserted into the region encoding the Dof DNA-binding domain in the *dof5.8-1* allele, this allele is a null allele. In *dof5.8-2*, T-DNA was inserted into the C-terminal region flanking the N-terminal Dof domain, suggesting that the product of this allele may retain its DNA-binding activity (Fig. 4A). However, because of the reduced transcript level in *dof5.8-2*, it is probably a loss-of-function allele (Fig. 4A). Although neither allele exhibited an apparent phenotype, it was found that they enhanced the phenotypes of a weak allele of *mp*, *arf5-2*, including abnormal root and cotyledon development. Seedlings of the *mp* mutants are rootless and often have only one cotyledon (Berleth and Jürgens, 1993). The penetrance of both phenotypes is low in the *arf5-2* allele (Donner *et al.*, 2009; Rademacher *et al.*, 2011). In an experiment using a population from the *MP/arf5-2* parent plant, 3.4% of the *arf5-2* seedlings were rootless, as 21 seedlings out of 614 showed the phenotype (Fig. 4B). Considerably larger numbers of seedlings were found to be rootless when populations from the parent plants that are homozygous for the *dof5.8* mutation and heterozygous for the *arf5-2* allele were investigated: 17.1% of the *dof5.8-1 arf5-2* population and 9.7% of the *dof5.8-2 arf5-2* population were rootless. Since the result of genotyping indicated that the percentage of *arf5-2* homozygotes was mostly the same (~20%) in these three populations (Table 1; Supplementary Fig. 1 at *JXB* online), it was concluded that *dof5.8* mutations increased the penetrance of the rootless phenotype of *arf5-2*.

**Fig. 5.**
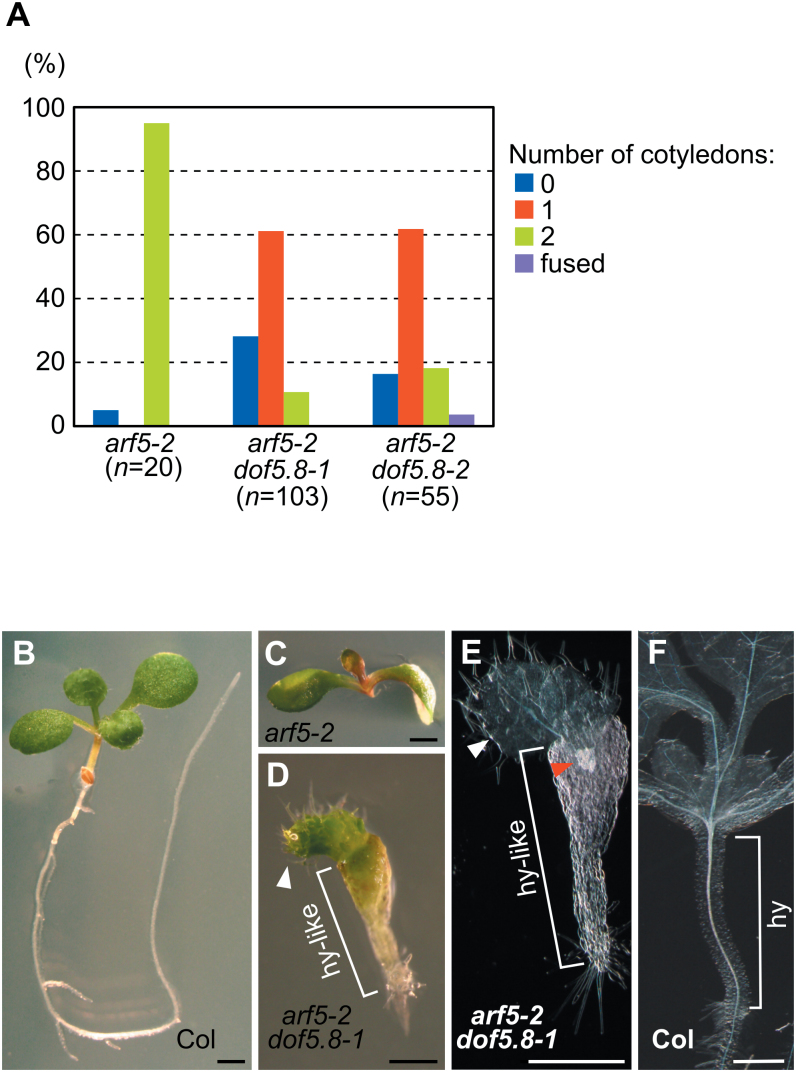
The synergistic effects caused by *dof5.8* and *arf5-2* mutations. (A) The number of cotyledons in rootless seedlings of *arf5-2* and *arf5-2 dof5.8* mutants. (B–D) Images of 7-day-old wild-type (B), *arf5-2* (C), and *arf5-2 dof5.8*–*1* (D) seedlings. (E, F) Cleared images of *arf5-2 dof5.8-1* (E) and Col (F) seedlings. White and red arrowheads in (D, E) indicate true leaves and vascular elements, respectively. ‘hy-like’ and ‘hy’ in (D–F) indicate a hypocotyl-like structure and hypocotyl, respectively. Scale bars=1mm in (B–D) and 0.5mm in (D–F).

Another effect of *dof5.8* mutations in the *arf5-2* mutant was also found. Most of the rootless *arf5-2* seedlings (95%) possessed two cotyledons, while far fewer seedlings of rootless *arf5-2 dof5.8-1* (10.7%) and *arf5-2 dof5.8–2* (18.2%) seedlings possessed two cotyledons (Fig. 5A). Furthermore, considerable numbers of the double mutant seedlings had no cotyledons, although such a phenotype was rarely seen in the single *mp* mutants. The cotyledon-less seedlings of the double mutants always had a fat hypocotyl-like structure, which was topped with true leaves with trichomes but did not include developed vascular elements (Fig. 5D, E). This result suggests that an interaction between *arf5-2* and *dof5.8* mutations influenced embryonic development and thus formation of cotyledons.

### Effects of *dof5.8* mutations on vascular patterning in the *arf5-2* mutant

**Fig. 6.**
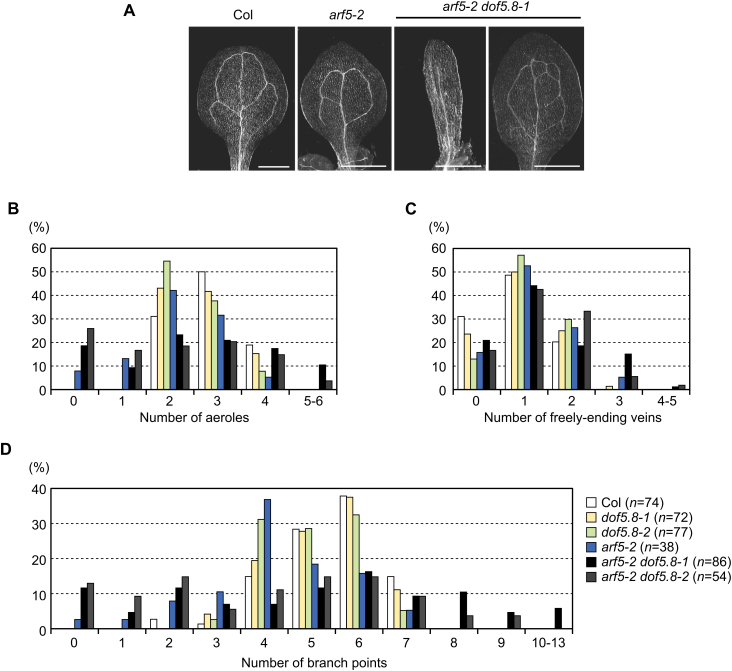
The vascular patterns of *arf5-2 dof5.8* cotyledons. (A) Representative images of the vascular pattern of cotyledons of wild-type (Col), *arf5-2* mutant, and *arf5-2 dof5.8-1* double mutant seedlings. Scale bars=1mm. (B–D) The percentage of cotyledons with the indicated number of aeroles (B), freely ending veins (C), and branch points (D). Seeds from plants heterozygous for *arf5-2* in the wild-type, *dof5.8-1* homozygous or *dof5.8-2* homozygous background were sown, and rootless seedlings were used in this analysis. (This figure is available in colour at *JXB* online.)

Vein patterns of the double mutants were also analysed, and it was found that the *dof5.8* mutation modulates the vascular pattern of *arf5-2* cotyledons. Vascular patterns in the cotyledons of wild-type *A. thaliana* are relatively simple and invariant, with the secondary veins delimiting the two upper aeroles and the zero to two lower aeroles (Fig. 6A). The majority of *arf5-2* cotyledons showed vascular patterns similar to those of the wild type (Fig. 6A). However, a small portion (7.9%) had no aeroles due to incomplete formation of the secondary veins (Fig. 6B). The *dof5.8* mutations increased the ratio of cotyledons lacking aeroles (18.6% in *arf5-2 dof5.8-1* and 25.9% in *arf5-2 dof5.8-2*; Fig. 6B). However, at the same time, some *arf5-2 dof5.8* cotyledons showed more complex vascular patterns with extra aeroles (10.4% in *arf5-2 dof5.8-1* and 3.7% in *arf5-2 dof5.8-2*; Fig. 6A, B). The number of branch points in *arf5-2 dof5.8* cotyledons also a showed similar, broader distribution (Fig. 6D). The formation of extra aeroles and branch points was not observed in the wild type, or in *arf5-2* or *dof5.8* single mutants.

## Discussion

In this study, it was shown that MP regulates the expression of the Dof5.8 transcription factor gene through its direct binding to the *Dof5.8* promoter sequence. It was also shown that two *Dof5.8* mutations (*dof5.8-1* and *dof5.8-2*) influence abnormal root and cotyledon development and vascular patterning in the *arf5-2* mutant, although the effects of *dof5.8* mutations alone were not recognizable in the wild-type genetic background. These phenotypes, together with the evidence that the expression of *Dof5.8* is regulated by MP, suggest a genetic interaction between *MP* and *Dof5.8* in the developmental programme in *A. thaliana*.

### Provascular expression of *Dof5.8*

The results of the deletion and mutation analyses of the *Dof5.8* promoter and a ChIP analysis indicated the contribution of MP to the activity of the *Dof5.8* promoter in provascular cells (Figs 2, 3). Nevertheless, there are still questions as to the observed phenomenon. The early activity of the *Dof5.8* promoter was observed inside leaf primordia where DR5 activity is absent. Because *MP* mRNA is present within primordia at this stage (Wenzel *et al.*, 2007), the lack of DR5 activity may reflect the lack of the activity of MP protein and the regulation of the *Dof5.8* promoter by other transcription factors, although there is another possibility that this phenomena is due to the limitation of using this synthetic reporter. Furthermore, the results of deletion and point mutational analyses in protoplasts and *in planta* were not perfectly consistent with each another: the effect of deletion of the region from –1558 to –1301 was stronger than that of the M1 mutation (Fig. 3). On the other hand, the result of a ChIP analysis suggests that binding to the region from –1558 to –1301 is weaker than that to the region from –1301 to –1077. Therefore, *cis*-elements for other transcription factors that act co-operatively with or independently of MP may be present in this region. Alternatively, MP might bind to two 5’-TGTC-3’ sequences in this region in addition to the first putative MP-binding site (site 1 in Fig. 3A), since the TGTC sequence, a part of the consensus sequence for MP binding (5’-TGTCNC-3’), functioned as an MP-binding site in the *Athb8* and *TMO7* promoters (Donner *et al.*, 2009; Schlereth *et al.*, 2010). Moreover, the mutations on four putative MP-binding sites (M1–M4) affected the Dof promoter activity differently in mesophyll protoplasts and leaf primordia. For instance, the M1 mutation appeared to reduce MP-dependent activation in mesophyll protoplasts and provascular cells to different extents (Fig. 3E, F). This fact also implies the possibility that other transcription factors besides MP are involved in the regulation of the expression of the *Dof5.8* promoter in provascular cells. Taken together, although the results indicated that the activity of the *Dof5.8* promoter in provascular cells is modulated by MP through the interaction with the region from –1558 to –1077, further analysis focused on this region would be necessary for complete understanding of the molecular mechanisms underlying the expression of *Dof5.8* in provascular cells.

### The role of *Dof5.8* in cotyledon and root formation, and vein patterning

The *dof5.8* mutations enhanced the effect of the *mp* mutation on embryonic root and cotyledon development, consistent with the expression of *Dof5.8* in embryos as well as in the immature veins of young leaves (Konishi and Yanagisawa, 2007). The *dof5.8* mutations increased the penetrance of the rootless phenotype of *arf5-2*, suggesting that, when the activity of MP is compromised, *Dof5.8* becomes critical for embryonic root formation. Such an auxiliary role to that of MP was also reported for ARF6 (Rademacher *et al.*, 2011). The *dof5.8* mutations also produced a synergistic effect with the *mp* mutation on cotyledon development, which resulted in cotyledon-less seedlings (Fig. 5). Although such a severe phenotype has rarely been reported for any single *mp* alleles, the combination of *mp* with a gain-of-function allele of *BDL*, or with the *nph4* mutation produces cotyledon-less seedlings (Hamann *et al.*, 1999; Hardtke *et al.*, 2004). BDL is an inhibitor of ARFs including MP, and *NPH4* encodes ARF7. Therefore, a more severe defect in auxin response during embryonic development could cause such cotyledon-less seedlings. The fact that *dof5.8 mp* double mutants showed such a severe phenotype implies that *Dof5.8* is associated with the auxin- and MP-induced developmental programme during embryogenesis.

In contrast to the effects in embryonic development, *dof5.8* mutations exert both positive and negative effects on vascular formation in leaf primordia of the *arf5-2* mutant. The vein patterns of cotyledons in both the wild type and the *arf5-2* mutant are relatively invariant, whereas the vein patterns of the *arf5-2 dof5.8* mutants exhibited larger variation, with both reduced and increased complexity in vein pattern (Fig. 6). Although this phenomenon is interesting, it is difficult to explain it if *Dof5.8* merely plays an auxiliary role to *MP* in vascular formation in leaf primordia. Thus, the molecular basis of this is currently unclear. More detailed analysis of *dof5.8* mutants in combination with mutations within other *Dof* genes that are expressed in provasuclar cells or other genes downstream of MP, as well as the identification of target genes of Dof5.8 would be necessary to reveal of the role of Dof5.8 in vascular formation in leaves.

## Supplementary data

Supplementary data are available at *JXB* online.

Figure S1. Genotyping of segregating populations of the *arf5-2* mutant harbouring the heterozygous *arf5-2* allele and the mutants that are heterozygous for the *arf5-2* allele and homozygous for the *dof5.8* allele.

Table S1. Primer list.

Supplementary Data

## Abbreviations:

ARF: auxin response factor
BDL: BODENLOS
ChIP: chromatin immunoprecipitation
2,4-D: 2,4-dichlorophenoxyacetic acid
GUS: β-glucuronidase
IAA: indole-3-acetic acid
LUC: luciferase
MP: MONOPTEROS
MS: Murashige and Skoog
qRT–PCR: quantitative reverse transcription–polymerase chain reaction.
