# Supplementary material for: MONOPTEROS directly activates the auxin-inducible promoter of the Dof5.8 transcription factor gene in Arabidopsis thaliana leaf provascular cells
Source: J Exp Bot. 2014 Oct 21;66(1):283–91. doi: 10.1093/jxb/eru418 (PMC4265163; doi:10.1093/jxb/eru418)
Supplement: Supplementary Data [file supp_66_1_283__index.html]

MONOPTEROS directly activates the auxin-inducible promoter of the Dof5.8 transcription factor gene in Arabidopsis thaliana leaf provascular cells — MONOPTEROS directly activates the auxin-inducible promoter of the Dof5.8 transcription factor gene in Arabidopsis thaliana leaf provascular cells — MONOPTEROS directly activates the auxin-inducible promoter of the Dof5.8 transcription factor gene in Arabidopsis thaliana leaf provascular cells — Supplementary Data 

# MONOPTEROS directly activates the auxin-inducible promoter of the Dof5.8 transcription factor gene in *Arabidopsis thaliana* leaf provascular cells

## Supplementary Data

Data files

**Files in this Data Supplement:**

- Supplementary Data - Supplementary Data
