# Supplementary material for: MONOPTEROS directly activates the auxin-inducible promoter of the Dof5.8 transcription factor gene in Arabidopsis thaliana leaf provascular cells
Source: J Exp Bot. 2014 Oct 21;66(1):283–91. doi: 10.1093/jxb/eru418 (PMC4265163; doi:10.1093/jxb/eru418)
Supplement: Supplementary Data [file supp_eru418_jexbot131698_file001.pdf]

Table S1. Primer list.

Gene	AGI number	Sequence
**qRT-PCR**		
Dof5.8, Fig. 4 (a)	At5g66940	AGGAGGATCTGTTGCGATTCC<br>CGAGGACAAGAAAGCTGCTC
Dof5.8, Fig.2 and Fig.4(b)	At5g66940	AAGCCGGAACTTTCCCTTAC<br>AGAGCTGAAACCGCCATAGA
Dof5.8, Fig. 4 (c)	At5g66940	TGGCAGTTTGAAGGTCAAGA<br>GATTGAAACCCTCCGATGAA
UBQ10	At4g05320	GGCCTTGTATAATCCCTGATGAATAAG<br>AAAGAGATAACAGGAACGGAAACATAGT
**ChIP-qPCR**		
a (-1492 to -1322)	At5g66940	CAGAGAGGAAAAGGACAAAAAGAAT<br>TTCACACCAAAAAGCATCCAACATT
b (-1268 to -1159)	At5g66940	TTACCCGACAACCTTCGCTTC<br>ACAGGAAAGAAGAGAGGGTAGTAAT
ATHB8	At4g32880	GAAAGGAAGGCTAAACGAATTTGC<br>GTGTCGGGCTGTGTTGAAAAG
UBQ10	At4g05320	CAAATTCCCTCCCTTTAAGCACC<br>AACTTATCCGGTCCTAGATCATCAG
**Cloning**		
MP[a]	At1g19850	CTACCATGGCTTCATTGTCTTGTGTTGAAGAC<br>GTAAGGCCTTGAAACAGAAGTCTTAAGATCGTTAA
BDL[a]	At1g04550	GTGCCATGGGTGGTGTGTCAGAATTGGAG<br>GTGAGGCCTAACAGGGTTGTTTCTTTGTCTATC
Dof5.8 promoter (M1)	At5g66940	TGTTGTGAGAAGTATGTGCCAAGAC<br>GAGTCTCCAAAATGTTGGATGCT
Dof5.8 promoter (M23)	At5g66940	GTTTTGTGAACTCTGTGAAGCGAAGGTTGTCGGGTAAAG<br>GAATCTCTTCACAGAGTTTTGACTGTTTTTAGCCTCCCCA
Dof5.8 promoter (M1234)	At5g66940	TGTAATGTGATTTGTTCCACAGGAAAG<br>GTGACTCTCTCTCTCTACCTTAAAG
**Genotyping of T-DNA lines**		
a	At1g19850	CATCTTTCCCTAGTATGGCTTCGG
b	At1g19850	CACTCATCTGCTGGACCTCA
c	At1g19850	TGGTTTCTCCTCTACCAGTTGGAG
d	At5g66940	CAGCCATGGGCATGCCTTCTGAATTCAGTGAATC
e	At5g66940	GCCAGGCCTCGCTACGTAGTCTCCAGACACGA
SALK_LBb1	-	GCGTGGACCGCTTGCTGCAACT

[a]Restriction sites for cloning are underlined.

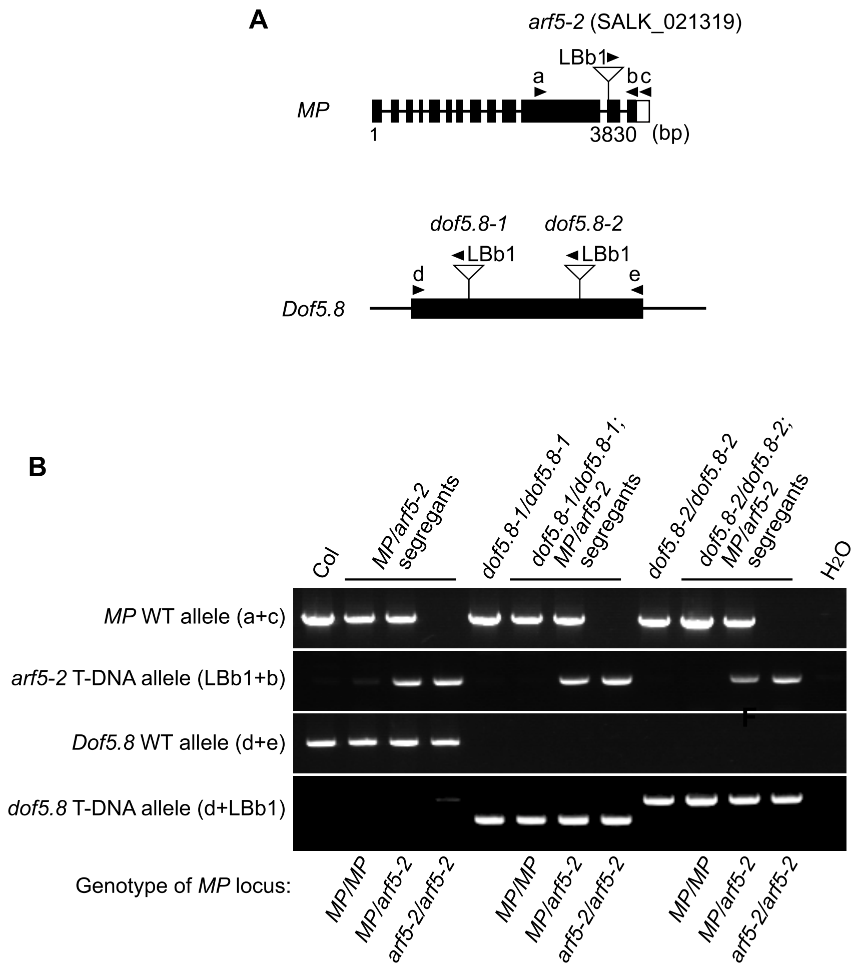

Figure S1. Genotyping of segregating populations of the *arf5-2* mutant harbouring the heterozygous *arf5-2* allele and the mutants that are heterozygous for the *arf5-2* allele and homozygous for the *dof5.8* allele. (A) Positions of primers used in genotyping. Sequences of the primers are shown in Table S1. (B) A representative image of the results of genotyping.
